# Quality of intrapartum care: direct observations in a low-resource tertiary hospital

**DOI:** 10.1186/s12978-020-0849-8

**Published:** 2020-03-14

**Authors:** Natasha Housseine, Marieke C. Punt, Ali Gharib Mohamed, Said Mzee Said, Nanna Maaløe, Nicolaas P. A. Zuithoff, Tarek Meguid, Arie Franx, Diederick E. Grobbee, Joyce L. Browne, Marcus J. Rijken

**Affiliations:** 1 Division Woman and Baby, University Medical Centre Utrecht, Utrecht, Netherlands; 2 Julius Global Health, Julius Centre for Health Sciences and Primary Care, University Medical Centre Utrecht, Huispost nr. STR 6.131, P.O. Box 85500, 3508 Utrecht, Netherlands; 3 Department of Obstetrics and Gynaecology, Mnazi Mmoja Hospital, Zanzibar, Tanzania; 4 School of Health and Medical Science, State University of Zanzibar (SUZA), Zanzibar, Tanzania; 5 Global Health Section, Department of Public Health, University of Copenhagen, Copenhagen, Denmark

**Keywords:** Labour, Obstetrics, Guidelines, Foetal monitoring, Intermittent auscultation, Low resource, Developing countries

## Abstract

**Background:**

The majority of the world’s perinatal deaths occur in low- and middle-income countries. A substantial proportion occurs intrapartum and is avoidable with better care. At a low-resource tertiary hospital, this study assessed the quality of intrapartum care and adherence to locally-tailored clinical guidelines.

**Methods:**

A non-participatory, structured, direct observation study was held at Mnazi Mmoja Hospital, Zanzibar, Tanzania, between October and November 2016. Women in active labour were followed and structure, processes of labour care and outcomes of care systematically recorded. Descriptive analyses were performed on the labour observations and compared to local guidelines and supplemented by qualitative findings. A Poisson regression analysis assessed factors affecting foetal heart rate monitoring (FHRM) guidelines adherence.

**Results:**

161 labouring women were observed. The nurse/midwife-to-labouring-women ratio of 1:4, resulted in doctors providing a significant part of intrapartum monitoring. Care during labour and two-thirds of deliveries was provided in a one-room labour ward with shared beds. Screening for privacy and communication of examination findings were done in 50 and 34%, respectively. For the majority, there was delayed recognition of labour progress and insufficient support in second stage of labour. While FHRM was generally performed suboptimally with a median interval of 105 (interquartile range 57–160) minutes, occurrence of an intrapartum risk event (non-reassuring FHR, oxytocin use or poor progress) increased assessment frequency significantly (rate ratio 1.32 (CI 1.09–1.58)).

**Conclusions:**

Neither international nor locally-adapted standards of intrapartum routine care were optimally achieved. This was most likely due to a grossly inadequate capacity of birth attendants; without whom innovative interventions at birth are unlikely to succeed. This calls for international and local stakeholders to address the root causes of unsafe intrafacility care in low-resource settings, including the number of skilled birth attendants required for safe and respectful births.

## Plain English summary

Every year around the world, 2.1 million babies die in the womb (stillbirths) and another 2.6 million die within 28 days of birth (neonatal deaths). About half of these deaths are associated with problems that occur during birth in resource-poor settings.

In this study, we assessed the quality of labour care at Zanzibar’s tertiary hospital, Tanzania. We directly observed and carefully recorded the care given to 161 women throughout birth and compared our findings to the local clinical guidelines of care at birth.

In this busy hospital, care during birth was provided by young nurses, midwives and doctors with little direct supervision from seniors. Conditions were difficult for both staff and pregnant women. Labour and delivery took place in an open and crowded labour ward. Each midwife took care of four women in labour simultaneously which resulted in insufficient support during birth.

Monitoring was not optimally performed according to the locally-tailored guidelines. For example, the baby’s heart was monitored every 105 min on average instead of the recommended 60 min. Staff, however, increased monitoring when certain problems were detected, such as when the baby’s heart was not beating normally, when progress of labour was slow and when oxytocin was used to increase contraction. Putting up a screen for privacy was done in about half of all vaginal examinations. In most cases, women were not informed of the findings after an examination. Main reasons for birth attendants being unable to follow their clinical guidelines were that they are too few. Thus an investment in sufficient and competent workforce in such labour wards is crucial.

## Background

An estimated 300,000 maternal deaths and five million perinatal deaths occur yearly worldwide, with > 98% in low- and middle-income countries (LMICs) [1]. Although childhood mortality has been reduced significantly, Millennium Development Goal 4 was not met in Sub-Saharan Africa, as neonatal deaths went mostly ignored and now make up 40% of under-5 mortalities [1, 2]. Almost half the number of stillbirths and 23% of neonatal deaths in LMICs are intrapartum-related, in contrast to high-income settings [3, 4]. Hence, ending intrapartum deaths by improved quality of intra-facility care is pivotal [5]. Yet, in Sub-Saharan Africa, the increasing numbers of facility-based deliveries, have not resulted in better intrapartum care and progress to improve perinatal health outcomes is slowest [6–8]. Instead, in many facilities, international standards of intrapartum care have become more difficult to implement in the day-to-day reality. As found in our hospital, after unrealistic international guidelines were adapted to better suit the local resource-limited reality, significant improvements were observed in quality of care, stillbirths were reduced by one-third and the number of neonates with birth asphyxia nearly halved (Box 1) [9].

Quality of intrapartum care has mostly been assessed by retrospective analysis of existing medical records [10]. However, written records (e.g. partographs) in low-resource settings are often incomplete, missing or inaccurate [10], and therefore might not reflect the actual care. The gold standard for clinical quality assessment is direct observation that captures the real-life experiences and behaviour of birth attendants; yet, they are rarely used [10, 11]. Similarly, few studies have assessed the adequacy of intrapartum foetal monitoring in low-resource settings [12–16]. This study used continuous direct observation to assess the quality of intrapartum care, with a specific focus on foetal monitoring and the structural requirements to delivering intrapartum quality care.

## Methods

### Study design

This was a prospective study consisting of labour observations at the maternity ward of Mnazi Mmoja Hospital (MMH) in Zanzibar, the United Republic of Tanzania, from October to November 2016. The study adhered to a pre-determined protocol (Additional file 1) and STROBE standards of reporting [17]. This manuscript is part of the larger PartoMa Project initiated in 2015 to improve quality of care (Box 1) [9].

### Setting

**Fig. 1:**
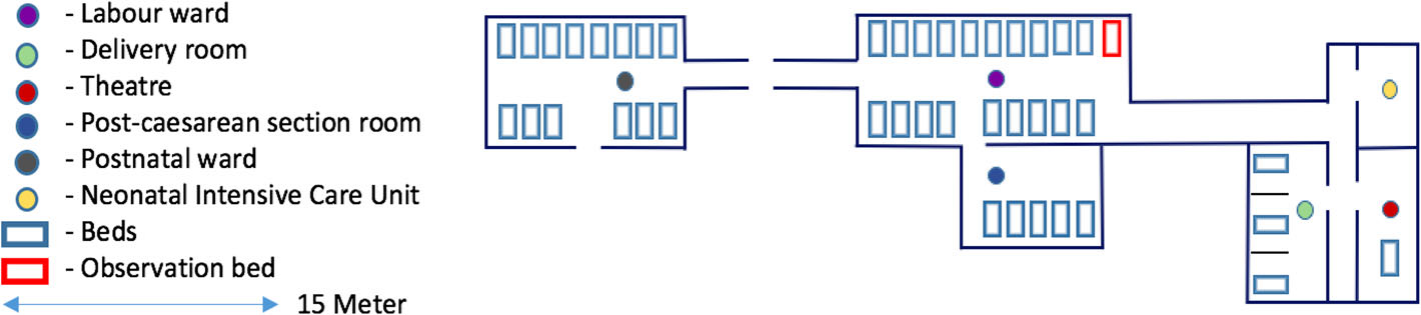
Layout of the maternity unit of Mnazi Mmoja Hospital (MMH) in Zanzibar, the United Republic of Tanzania (2016)

About 12,000 annual deliveries are assisted at MMH, and it is the only tertiary hospital on the Zanzibar archipelago [12]. At the time of the study, the labour unit consisted of an admission room, a one-room labour ward with 19 beds, a three-bed delivery room, two postnatal rooms, and one theatre (Fig. 1). For privacy reasons, women were not allowed to have a companion during childbirth in the busy labour ward. Skilled birth attendants (SBAs) comprised 27 nurse-midwives (diploma-level in nursing, except three seniors with university-level degree), 13 resident doctors (general doctors) and six intern doctors (total *n* = 46) who together provided routine labour care and comprehensive emergency obstetric and newborn care. On average, six to eight nurse-midwives and five doctors were allocated to the maternity unit in the morning shifts and a total of six SBAs were in the remaining shifts (including weekends). These were the main roles of nurse-midwives: assessment on admission; intrapartum and postpartum care including supportive care, routine monitoring, administration of medication, vaginal deliveries, and perineal repair; maintenance of ward hygiene, and delivery sets. The roles of the doctors involved providing labour and postpartum care to high risk women and complicated deliveries and performing obstetric and gynaecological operations. Direct supervision was provided by two senior midwives and two senior (visiting) doctors, only during morning shifts. In addition, there were three obstetricians who could be consulted and called for emergencies (Additional file 2, cadre definitions). Standards for labour management was according to the peer-reviewed PartoMa Pocket Guide version 1.2 [9]. where, in collaboration with the SBAs, international guidelines had been tailored to the situation at the hospital, including reductions in information load, ambiguity, and frequency of clinical assessments (Additional file 3, comparison of FHRM recommendations) [9]. The stillbirth rate was 39 per 1000 total births, of which 38% occurred during intra-hospital care [9].

### Participants

The first women to reach active phase of labour (≥ 4 cm cervical dilatation), either at admission or already in the ward, were selected for inclusion. Exclusion criteria were absent foetal heart rate (FHR) on admission, elective or emergency caesarean section (CS) decided immediately after admission, and known congenital foetal anomaly incompatible with life. Shifts of inclusion were planned beforehand to ensure representation of morning, evening, and night shifts throughout the week. Labours were observed until delivery or diagnosis of intrauterine foetal death.

### Ethical approval

Approval from Mnazi Mmoja Hospital and Zanzibar Medical and Research Ethical Committee the local ethics committee (ZAMREC) was obtained (ZAMREC/0002/May/016). Written informed consent in Swahili was sought from all participating women. The aim of the study and the role of observers were introduced to all staff before commencing the study (not to cause blame or undermine the staff’s devotion to labouring women). In case observers had concerns about the safety of a woman, they could express these concerns to the staff on duty and a senior staff could be called for assistance if needed.

### Variables

**Fig. 2:**
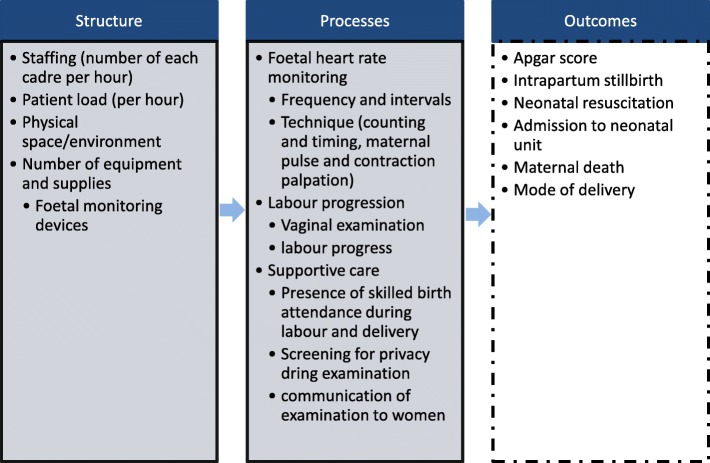
Variables measured in the study, related to structure and processes of care, by the Donabedian framework

The study qualitatively described and quantified structural indicators and processes of intrapartum care, as described in the Donabedian model [18]. It focused on the aspects of intrapartum monitoring and supportive care (Fig. 2). In addition, the study determined adherence to the local FHRM guidelines (in terms of frequency and technique) [9], and the effect of five pre-identified predictors on FHRM frequency: pregnancy risk status (Box 2); occurrence of intrapartum risk events (Box 2) [19]; parity (nulliparity and multiparity); SBA’s years of experience with maternity care; and shift of inclusion. Predictors were adopted from the NICE [19] and local PartoMa guidelines, and from the hypothesis that they would alter the frequency of observations and/or the quality of care. Adherence to FHRM guidelines meant that the number of FHRMs was at least equal to the expected frequency. Expected frequencies of FHRM for low-risk labours, high-risk labours, and non-reassuring/abnormal FHR were set at 60, 30, and 15 min, respectively [9]. These were based on first stage of labour guidelines, because the start of second stage was often unknown. Other variables recorded consisted of socio-demographic characteristics, risk assessment variables (Box 2), cervical dilatation, and FHR on admission (Additional file 4).

### Data sources/measurement

The five observers were not qualified to act as birth attendants. One of the observers was a foreign final year medical student. The other four observers were newly graduated local medical students who had recently completed their studies and awaiting approval to start clinical internships. Therefore, none of the observers were permitted to provide medical care in this setting. Two observers per shift, both located in the labour room, conducted non-participatory direct observations. They recorded all care provided to each woman and performed hourly counts of the structural indicators (Fig. 2). When a woman was taken to the delivery room or theatre, one observer followed her until birth. Sociodemographic characteristics, risk assessment variables, assessments on admission and birth outcomes were collected using the women’s records (antenatal card and hospital file). Two data collection forms were created (Additional file 4), pilot tested, and used for the systematic recording [18]; one for processes of care provided per woman as well as cadre of staff who provided the care and birth outcomes, and one for hourly counts of number of birth attendants, labouring women, and functioning FHRM devices. Both forms provided space for free-text description of e.g. supportive care (staff’s and women’s behaviour, presence of SBA during delivery), decision-making, medical treatment, and physical space/environment.

### Bias

Observer bias was a major concern. Observers could have underreported assessments made by SBAs or could have been involved in providing labour care. To minimise these biases, they were trained to recruit women into the study, make objective structured assessments with minimal intervention in care provision, and record their observations on the forms. They worked in pairs and observed a maximum of eight labouring women at a time. Also, the presence of observers could have altered the behaviour and improved performance of the SBAs (the Hawthorne effect) [20]. However, this effect was likely to be minimal because the labour ward was extremely busy. Also, observations were made from an adequate distance, rather than by following the SBA.

### Study size

The a priori sample size was estimated to be 150 labours plus 10% to account for potential hindsight exclusion based on a maximum of five predictors, with the aim to detect a single predictor with an assumed effect size of a risk ratio of 1.3, power of 80% and alpha of 5%.

### Analysis

First, descriptive analyses were conducted on women’s characteristics and number of staff, women, care provision, and FHRM devices. Means were reported with standard deviations (SDs), medians with interquartile ranges (IQRs), and frequencies with percentages. Then, the proportion of observations adhering to local FHRM guidelines, privacy standards and communication practices were calculated using the following formula: number of observations divided by number of expected observations, given the duration of labour. Four intervals were calculated: between two adjacent FHRMs, vaginal examinations, contractions and the last FHR-to-delivery interval. Differences between work shifts (day/evening/nights) were tested using one-way-ANOVA for the number of staff, equipment and labouring women and chi-square test for composite adverse perinatal outcomes (i.e. stillbirths and Apgar score < 7 at 1 min). Furthermore, univariate and multivariate generalized linear models for Poisson distributions were performed with the number of FHRMs for each woman as an outcome to estimate the effect of the above-mentioned pre-selected predictors on local guidelines adherence. In the multivariate analysis, the predictor intrapartum risk event was dichotomised. Results from the analysis were reported as rate ratios (RRs) with 95% confidence intervals and corresponding *p*-values. For this study, a RR may be interpreted as the increase in the number of FHRMs either compared to a reference category or when a continuous predictor increases by one unit. The set frequency of FHRM was hourly and women with a total pre-delivery observation of less than one hour were excluded from this analysis. The statistical software used was SPSS version 23, and SAS 9.4. Free-text comments were used to supplement quantitative findings on human resources, physical space, equipment, monitoring, and supportive care.

## Results

### Participants and birth outcomes

The majority of the 161 women observed (95.0%, *n* = 153) came directly from home, at term (39 (±2.8) weeks), and with a median cervical dilatation of five (IQR: 3–7) cm. Inclusions were spread evenly between morning, evening, and night shifts (34.2%, *n* = 55; 30.4%, *n* = 49; and 35.4%, *n* = 57, respectively). Fifty-one women (31.7%) were classified as high-risk on admission; the most common risk factors being hypertensive disorders (10.5%, *n* = 17), prematurity (8.1%, *n* = 13), breech presentation (6.8%, *n* = 11, including two singletons and one twin pregnancy diagnosed close to delivery), previous uterine scar (5.6%, *n* = 9) and grand multiparity (5.6%, n = 9). In addition, 38.2% (*n* = 42/110) of the remaining labouring women experienced one or more intrapartum risk events. Of the 42 (26%) labours augmented with oxytocin, two thirds (*n* = 28) had crossed the action line of the partograph (i.e. poor progress). There were no maternal or neonatal deaths before hospital discharge, but there were four stillbirths (2.4%) and 23 (14.3) babies with Apgar score less than seven at one minute (Table 1). There was no statistical significance in composite perinatal outcomes between shifts of delivery (*p* = 0.70).

**Table 1:** Pregnancy and labour characteristics and outcomes

Parameter	N(%)^*^
Maternal age in years, mean (SD)	26.4 (6.3)
Parity	
Nulliparous	86 (53.4)
Multiparous	75 (46.5)
Singleton	157 (97.5)
Twin	4 (2.5)
Presentation	
Cephalic	150 (93.2)
Breech	11 (6.8)
Number of antenatal care visits, mean (SD)	3.7 (1.3)
Referral pathway	
From home	153 (95.0)
Referral from another health facility	8(5.0)
Known gestational age by last menstrual period/Ultrasound	72 (44.7%)
Gestational age by last menstrual period/ultrasound, in weeks, mean (SD)	39.0 (2.8)
Fundal height in cm, mean (SD)	34.4 (3.5)
Cervical dilatation on admission	
< 4 cm cervical dilatation	51 (31.5)
≥ 4 cm cervical dilatation	110 (67.9)
Cervical dilatation at admission in cm, median (IQR)	5 (3–7)
Duration of observation in minutes, median (IQR)	290 (135–570)
Risk category on admission	
Low-risk	110(68.3)
High-risk	51 (31.6)
Intrapartum risk events	
None	92 (57.1)
Meconium-stained liquor	20 (12.4)
Abnormal FHR	8 (5.0)
Oxytocin use (including induction of labour)	51 (31.7)
Maternal pyrexia	2 (1.2)
Action line on partograph crossed	37 (25.0)
Mode of Delivery	
Spontaneous vaginal delivery (SVD)	134 (83.2)
Vacuum	5 (3.1)
Caesarean Section	21 (13.0)
SVD and Caesarean Section (twin)	1 (0.6)
Delivery Location	
Labour ward	90 (55.6)
Delivery Room	46 (28.4)
Theatre	24 (14.9)
Delivery Room and Theatre (twin)	1(0.6)
Perinatal outcomes (Total number of neonates = 165)	
Birthweight, grams, mean (SD)	3152.6 (535)
Apgar score < 7 at 1 min**	23 (14.3)
Apgar score < 7 at 5 min^**^	4 (2.5)
Resuscitation^**^	9 (5.6)
Admission to neonatal unit**	13 (8.3)
Intrapartum stillbirth (i.e. presence of foetal heart rate on admission)***	4 (2.4)
Neonatal deaths before discharge	0 (0.0)

^*^Unless otherwise specified values are given as number (percentage), ^**^of the live births, *** one stillbirth was delivered macerated.

### Structure: context in which care was provided

#### Human resources

The majority of nurse-midwives and doctors (89%, *n* = 41/46) had a maximum of five years’ experience in labour care (< 1 year: 41%, *n* = 19/46; 1–5 years: 48%, *n* = 22/46; > 5 years: 11%, n = 5/46). Observation at hourly intervals showed an average of nine labouring women and two to three nurse-midwives in the labour and delivery rooms throughout the day (nurse/midwife-to-labouring-women ratio of 1:4). Nurse-midwives performed all admission assessments, conducted 32.6% (134/411) of intrapartum monitoring and the majority of vaginal deliveries (67.1%, n = 94/140) (Table 3). The resident doctor on duty also monitored women in labour (38.9% of examinations, *n* = 160/411) and handled gynaecological and obstetric emergencies such as CS, obstetric haemorrhage and eclampsia. Sharing of information within cadres occurred during handover rounds, and two clinical meetings in the morning shift in which mainly doctors attended. Several SBAs regularly worked at a continuous pace and attended to women; while others showed signs of exhaustion, such as decreased work speed, resting for long periods, sleeping during night shifts, and uncourteous behaviour towards women and colleagues, only offering help when women became too loud or in the presence of a senior. Students and cleaners also shared tasks; they assisted during deliveries, offered psychological support, helped women with food and facilitated communication between women, SBAs and their families who were restricted access to the maternity unit.

#### Equipment and supplies

Partograph copies were available throughout the study period and were used in at least 87% (*n* = 140) of labours; the remaining were either lost after delivery (9.3%, n = 15) or not used at all (3.7%, n = 6). Difficulties encountered with FHRM devices were their scarcity or misplacement (Table 2), unavailability of gel for hand-held Dopplers and ultrasound, and non-functioning hand-held Dopplers (*n* = 6). Other intermittent shortages included lack of essential medication (e.g. antihypertensive), and supplies for vacuum extraction, CS and normal delivery sets.

**Table 2:** Cadre of staff, women and equipment available per shift

	Type of shift			p-value
	Morning	Evening	Night	
Number of birth attendants per hour	5.9(1.35)	4.0(1.2)	3.4(0.82)	0.001*
Nurse-midwives	2.6(0.70)	2.2(0.55)	2.2(0.44)	0.36
Resident doctors	1.4(0.60)	1.0(0.64)	0.8(0.4)	0.18
Intern doctors	1.2(0.68)	0.6(0.48)	0.4(0.46)	0.02*
Seniors (doctor)	0.8(0.63)	0.2(0.26)	0.0(0.07)	0.002*
Foetal heart rate devices per shift	7.8(1.83)	6.8(1.69)	7(1.55)	0.37
Pinard stethoscope	2.2(1.0)	2.7(0.68)	2.5(0.84)	0.47
DeLee stethoscope	2.7(1.0)	1.8(0.79)	2.3(1.2)	0.25
Hand-held Doppler	1.1(1.05)	0.5(1.3)	0.2(0.41)	0.23
Mobile Ultrasound	2.0(0.0)	1.8(0.42)	2.0(0.0)	0.22
Labouring women per hour	9.0(2.92)	9.3(2.31)	8.6(2.25)	0.89

Values are given as mean (standard deviation)

*Significant level at 0.05: there were less senior doctors in the evening and night, and less intern doctors at night compared to morning shifts

#### Physical space

The single-room labour ward was noisy and crowded, and consisted of women in all stages of labour and post-delivery. The number of women typically exceeded the number of beds, thus women often shared and changed beds. Although specific areas of the room were reserved for high risk women and specific stages of labour, this localisation was not consistently used. Two thirds of vaginal deliveries took place in the shared labour ward (64.3%, *n* = 90/140), while 32.9% (*n* = 46/140) reached the three-bed delivery room that had more privacy (Table 1).

#### Processes of routine care delivery

527 provider-woman contact points were observed during labour and delivery, which included 411 examinations consisting of FHRM (*n* = 268), vaginal examinations (*n* = 326), and other forms of care (*n* = 116) (Table 3). Maternal blood pressure and/or temperature were measured at 180 of these time points. The median decision-delivery time interval for the 27 emergency operative deliveries was 60 (IQR: 25–81) minutes.

**Table 3:** Care and provider at each provider-to-woman contact point

Care provided	Nurse-Midwives	Residents	Intern	Visiting doctors	Multiple cadres	Others^f^	Total
FHR^a^ assessments	22(25.9)	34(40.0)	9(10.6)	17(20.0)	3(3.5)	0(0.0)	85
VE^a,b^	65(45.5)	59(41.3)	8(5.6)	3(2.1)	2(1.4)	5(3.5)	143^e^
FHR and VE^a^	47(25.7)	67(36.6)	23(12.6)	27(14.8)	15(8.2)	3(1.6)	183^e^
Other labour care^c^	43(37.1)	37(31.9)	13(11.2)	5(4.3)	9(7.8)	9(7.8)	116
Conducting delivery^d^	94 (58.4)	40 (24.8)	6 (3.7)	10 (6.2)	–	11(6.8)	161

Values are given as number (percentage) or number

Abbreviations: *FHR* Foetal Heart Rate, *VE* vaginal examination,

^a^ Alone or with other labour care

^b^ Only VE for first stage of labour included

^c^ This mostly consisted of: IV fluids/drugs administration, urinary catheterisation and blood pressure measurement

^d^ Only main person conducting delivery was recorded

^e^ One examination in which cadre was not recorded

^f^ Nurse students except for one delivery by a cleaner

#### Caring support

Labouring women were provided intermittent care collectively by the SBAs on duty. They often called for attention, especially during contractions and when they felt the need to push. This prompted examination and diagnosis of second stage of labour in the majority of women who delivered vaginally (95.0%, *n* = 133/140). In more than a quarter of women (27.9%, *n* = 39/140), support during second stage was not provided until ≤5 min before delivery; six of whom delivered unattended. Six of the 39 women were admitted with < 4 cm cervical dilatation and also went through the entire first stage of labour unobserved. Other actions associated with caring support, including use of a screen for privacy and communication during and after examinations (FHRM and/or vaginal examination) were conducted in 50.1% (*n* = 206/411) and 34.1% (*n* = 140/411) respectively. (Table 4).

**Table 4:** Adherence to local guidelines

	Adhered to local guidelines (frequency, %)		
	Low-risk	High-risk	Total
FHR monitoring	4/110 (3.6)	1/51 (2.0)	5/161 (3.1)
Maternal pulse palpation during FHR assessment	7/164 (4.2)	8/104 (7.7)	15/268 (5.6)
Timing of FHR with the clock	63/164 (38.4)	31/104 (29.8)	94/268 (35.0)
Contraction palpation during FHR assessment	24/164 (14.6)	19/104 (18.3)	43/268 (16.0)
Screen used for privacy	120/262 (45.8)	86/149 (57.7)	206/411 (50.1)
Communication after exam	85/262 (32.4)	55/149 (36.9)	140/411 (34.1)

Legend: *FHR* Foetal heart monitoring, frequency was calculated per labour. Maternal pulse, timing and contraction is for each FHR assessment. Communication and use of screen is calculated for each time point of examination (FHRM and/or vaginal examination)

#### Routine monitoring of labour progress and foetal heart rate

The median duration of labour observation was 290 (IQR:135–570) minutes in which the median frequency of FHRM was one (IQR: 0–3) and of vaginal examination two (IQR:1–3). The median interval between two vaginal examinations in the first stage of labour was 125 (IQR 56–225) minutes. In none of the cases, the strength and frequency of contractions were assessed by 10-min palpation per abdomen.

Two-thirds of FHRM were conducted with a DeLee or Pinard stethoscope (69.4%, *n* = 186/268; DeLee: *n* = 117, Pinard: *n* = 64, both: *n* = 5). Ultrasound was primarily used in 23.5%, (*n* = 63/268) of cases, while hand-held Doppler was rarely used (1.9%, *n* = 5/268). In the remaining cases, ultrasound, following use of stethoscope, was used to confirm FHR (5.2%, n = 14/268). In 37.9% (*n* = 61) of labours, FHRM was only recorded on admission. For labours with more than one FHRM (*n* = 254), the median interval between two FHRMs was 105 (IQR 57–160) minutes, with 30% (*n* = 76/254) of intervals within 60 min, and 42.5% (*n* = 108/254) beyond two hours. The median interval between the last FHRM and delivery was 87 (IQR:41–170) minutes (Table 5). Of all FHRMs observed, they were counted with a clock in 35.0% (*n* = 94/268), maternal pulse simultaneously palpated in 5.6% (*n* = 15/268), and contraction palpated in 16.0% (*n* = 43/268) of cases. The locally recommended one-hour frequency of FHRM was adhered to in 3.1% (*n* = 5) women throughout labour (Table 4). There was no difference in last FHRM to delivery intervals between morning, evening, and night shifts. The presence of intrapartum risk events led to an increase in the number of FHRMs observed, with a rate ratio (RR) of 1.33 (CI 1.11–1.64), when a non-reassuring/abnormal FHR was detected (RR 1.59; CI 1.19–2.13), oxytocin was used (RR 1.25; CI 1.02–1.54, and when labour crossed the action line on the partograph (RR 1.25; CI 1.00–1.56) (Additional file 5).

**Table 5:** Times intervals between foetal heart rate assessments and vaginal examination

	Median(IQR)*
Foetal heart monitoring (hours and minutes):	
FHR interval between admission and next FHR assessment**	162(70–261)
FHR interval	100(51–193)
≤ 15 min n(%)	23(9.1)
16 to ≤ 30 min n(%)	19(7.5)
31 to ≤ 60 min n(%)	34(13.4)
61 ≤ 120 min n(%)	70(27.6)
> 120 min n(%)	108(42.5)
Overall last FHR to delivery interval	87(41–170)
Last FHR to delivery interval: Morning	83(35–145)
Last FHR to delivery interval: Evening	84(45–162)
Last FHR to delivery interval: Night	98(36–216)
Vaginal Examination time intervals in first stage	125(56–225)
≤ 2 h n(%)	188(47.7)
≤ 4 h n(%)	127(32.2)
> 4 h n(%)	79(20.1)

* Unless otherwise specified, results are presented as median (IQR), **Excludes women admitted with < 4 cm cervical dilatation

**Box 1:** Summary of the PartoMa intervention at Mnazi Mmoja Hospital, Zanzibar

The PartoMa Project was initiated at Zanzibar’s tertiary hospital, Tanzania in January 2015. Its objective was to improve quality of care and perinatal outcomes. Skilled birth attendants were involved in focus groups discussions, adapting international labour management guidelines to better suit their local situation and participation in trainings. Prior to the PartoMa intervention, the stillbirth rate was 59 per 1000 total births (52% had positive foetal heart rate on admission) and the rate of Apgar score of ≤5 was 52 per 1000 live births. At the 12th intervention month, stillbirth rate had decreased to 39 per 1000 total births (relative risk 0.66, 95% CI 0.53–0.82; intra-hospital singleton stillbirths reduced from 28 to 15 per 1000 total births) and Apgar score ≤ 5 fell to 28 per 1000 live births (relative risk 0.53, 95% CI 0.41–0.69). This was associated with improved quality of care, including improved foetal heart rate surveillance (a reduction in median time interval from last FHR to delivery from 120 (IQR 60–240) to 74 (IQR 30–130) minutes), more judicial use of oxytocin and improved management of women with severe hypertensive disorders.

**Box 2:** Definitions of high-risk labours and intrapartum risk events

High-risk factors	Intrapartum risk events
Previous caesarean section Medical complications (e.g. hypertensive disorders, diabetes or fever) Grand-multiparity (> 4 previous deliveries) Prematurity (< 37 weeks) Post-term pregnancy (> 42 weeks) Prolonged rupture of membranes (> 24 h) Multiple pregnancy Breech presentation Meconium-staining of the liquor Abnormal vaginal bleeding	Oxytocin/misoprostol use Vaginal bleeding Maternal fever Non-reassuring/ abnormal foetal heart rate (Supplementary file 2) Meconium-staining of the liquor Cord prolapse

## Discussion

This direct observation study reports on intrapartum care provided to 161 women in a congested tertiary hospital in Sub-Saharan Africa. It shows suboptimal birth attendance and adherence to local and international guidelines on timely care, including surveillance of the woman’s vital signs, FHR, and labour progress. Substantial findings of this study were the discontinuous care. Lack of support and respectful care were reflected by a significant absence of communication, privacy, and support at the time of delivery. Structural challenges observed were high workload compared to staff numbers, an unconducive environment and scarcity of monitoring devices. However, despite this congested and unconducive environment, SBAs showed ability to provide evidence-based triage; they prioritised the monitoring of women who were recognised to have an intrapartum risk event (non-reassuring/abnormal FHR, oxytocin use, and crossing the action line) leading to a significant increase in frequency of FHR assessments.

Lack of attendance and delay in diagnosing second stage of labour in the majority of women was the rationale behind our conservative approach to determine FHRM guidelines adherence. We only compared FHRM to first stage expected frequencies, which were lower than second stage frequency. Although this approach underreports the problem, the findings highlight the challenge of adhering to guidelines in these settings. In other resource-limited settings of Sub-Saharan Africa, randomised control trials on innovative FHRM devices failed to show improvement in perinatal outcomes due to non-adherence to FHRM international guidelines and obstetric response [21–24].

In this hospital, international guidelines were adapted to locally-acceptable minimum standards [9]. Compared to the situation before local guidelines were implemented, 20 months prior, there was sustained multiple improvements in monitoring: including higher FHRM frequency, notably in women with intrapartum risk events, more timely oxytocin use for augmentation, improved care for women with severe hypertensive disorders, and lower intrapartum stillbirth rates [9, 12]. An earlier study showed that these guidelines were positively viewed and were used by local staff [25]. However, they are still far from being adhered to optimally which present an ethical dilemma of whether such guidelines can further reduce frequency of assessments.

Inadequate number of health workers, as reflected in the hourly mid-wife-to-labouring women ratio, remained the major bottleneck to following internationally agreed evidence-based standards of care optimally. For example, with one nurse-midwife attending four women simultaneously, there is insufficient time to palpate contractions in accordance to international recommendations (every 30 min for 10-min duration), not to mention time for any other care or rest. Providing support throughout birth was thus an overarching challenge, notably of women in early labour who were not considered as yet eligible for routine intrapartum care. However, women in general, including in Sub-Saharan Africa, favour continuous birth companionship [26]. Evidence suggests that it is the most significant intervention during birth associated with positive effects on perinatal outcomes and women’s experience of birth [27]. As such the lack of support in this study, including in the second stage of labour, meant that women were likely to have had significant distress and negative birth experience. In addition to suboptimal care, this inevitably leads to a workforce with moral distress, burnout, and compassion fatigue hence, even less capable of giving compassionate care [28]. In order to cope with the high workload, resident doctors performed a significant part of routine intrapartum care that is usually conducted by midwives. However, this may have impaired the ability to provide obstetric care to the large number of high-risk women and further disrupted midwifery-led supportive care. The lack of adequately trained SBAs, in particular nurse-midwives, may have been exacerbated by lack of inter-professional collaboration and supervision of junior staff, as well as by inefficient organisation of space, workforce and tools [29]. Consequently, care remained severely suboptimal and the stillbirth rate persistently high, emphasising the need to first improve the basic structure of care. Achieving minimal standards of care would require efficient allocation of available resources, organisation, supervision and teamwork as well as an adequate increase in human resources. Moreover, birth companionship is a challenge in open and overcrowded labour wards. Efforts should also be placed on how e.g. relatives and traditional birth attendants may assist the overstrained skilled birth attendants in providing continuous support during labour and delivery.

### Strengths, limitations and ethical consideration

We here report the labour observation aspect of a broader work that included an ethnographic study and a locally co-created intervention to improve quality of labour care. Measurement of the quality of labour care is essential for identifying gaps, developing context-tailored interventions and monitoring of quality improvement processes. This unique systematic observation of intrapartum routine care overcomes the shortcomings of records-based studies in assessing quality of care. It allowed evaluation of the interactions between labouring women and their birth attendants, and the structural context care was provided in. The findings are supported by record-based quantitative PartoMa findings and also by mainly qualitative findings that showed similar suboptimal structural challenges to processes of care in numerous areas across Sub-Saharan Africa and other low resource-settings [12, 29–31]. However, observations are more resource-consuming than record-based assessments, hold a higher risk of the Hawthorne effect, and may pose ethical dilemmas about the non-participatory nature of the observer and their moral responsibility to participant safety in understaffed settings. Thus, a trade-off of biased results for participant safety may be necessary. As the observers were not qualified birth attendants, they expressed any concerns on patient safety, including imminent delivery, to the staff on duty and in emergency situations, they assisted the staff when requested. Observations show it was common practice for birth attendants to wait until signs of imminent delivery before attending to women in the second stage of labour. Thus a few women delivered unattended, as a result of preventable delays to respond to women’s call for help and not the mere absence of skilled birth attendants from the labour ward.

Limitations included the observer bias and Hawthorne effect described above. Also, the study aimed to measure and identify challenges, rather than detect differences in outcomes for varying quality of care. Hence, it was still unable to determine the effect foetal monitoring has, if any, on birth outcomes [16]. The results should then lead to adequately-powered studies including clinical auditing in other LMICs to estimate the quality of the intrapartum (foetal) monitoring and linking it to birth outcomes. Moreover, we were not able to determine the effect of staff experience and time of day on the quality of care as women were cared for by several birth attendants across multiple shifts. The admission assessment was retrieved from the patient file and not observed. Numerous findings on admission which included smaller-than expected fundal height, undiagnosed twins, breech presentation, and intrauterine foetal death until close to delivery indicate inadequate risk assessment and suggests admission time as an important point for improvement to explore.

## Conclusion

In this reality check of intrapartum care, the quality of basic routine care in a Tanzanian referral hospital remained unacceptable. It was not possible to provide respectful and safe care, and even to optimally follow locally adapted clinical guidelines, which took the local resources into account. This was particularly due to the disproportionate birth-attendant-to-labouring women ratio. Ensuring a safe and positive birth experience requires local stakeholders and international community to urgently address the structural barriers in Sub-Saharan Africa and invest in sufficient numbers of adequately trained and motivated staff for continuous support during labour.

## Supplementary information

**Additional file 1.** Study protocol

**Additional file 2.** Definition of various terms used in the study

**Additional file 3.** Comparison of foetal and contraction monitoring recommendations in international, national and local (PartoMa) guidelines

**Additional file 4.** Data collection sheets

**Additional file 5.** Predictors of the frequency of FHR monitoring

## Data Availability

The datasets used and analysed during the current study are available from the corresponding author on reasonable request.

## Abbreviations

CS: Caesarean section (CS)
FHR: Foetal heart rate
FHRM: Foetal heart rate monitoring
IQR: Interquartile range
LMIC: Low and middle-income countries
MMH: Mnazi Mmoja Hospital
NICE: National Institute for Health and Care Excellence
RR: Rate ratio
SAS: Statistical Analysis System
SBA: Skilled birth attendant
SD: Standard deviation
SPSS: Statistical Package for the Social Sciences
VE: Vaginal examination
