# Supplementary material for: Quality of intrapartum care: direct observations in a low-resource tertiary hospital
Source: Reprod Health. 2020 Mar 14;17:36. doi: 10.1186/s12978-020-0849-8 (PMC7071714; doi:10.1186/s12978-020-0849-8)
Supplement: Supplementary file 1 — Additional file 1. Study protocol [file 12978_2020_849_MOESM1_ESM.docx]

***Foetal heart monitoring in low-income countries*: *clinical observations.***

**Scientific Abstract**

Almost all (>98%) perinatal deaths occur in low- and middle-income countries (LMIC) and are related to suboptimal care and obstetric complications. Intrapartum-related complications account for a large proportion of neonatal death and childhood morbidity. The lack of skilled human resources in health care is a crucial bottleneck for the scale up of (cost-) effective evidence-based interventions to reduce perinatal mortality in low-income countries (LIC). [1-7].

This project will commence in the only referral hospital in Zanzibar, Tanzania. The aims of the overall project are to find novel strategies of intrapartum care in low resourced settings (ethical approval no: ZAMREC/0002/May/016).

The systematic review on *strategies and implementation of intrapartum foetal surveillance in LMIC* (registered on PROSPERO, CRD42016038679), provides an overview of the available literature on FCM in this setting. This part of the research project consists of two aims: a baseline assessment of quality of intrapartum care and a preparation for an analysis of the record keeping. This allows us to compare the intrapartum care via a criteria based audit in which current practice will be compared with local guidelines. The analysis of record keeping could determine the extent to which the records can be reliably used to assess quality of care in future studies.

**References**

1. Cousens S, Blencowe H, Stanton C, et al. *National, regional, and worldwide estimates of stillbirth rates in 2009 with trends since 1995: a systematic analysis*. Lancet 2011; 377: 1319–30.
2. Oestergaard MZ, Inoue M, Yoshida S, et al. *Neonatal mortality levels for 193 countries in 2009 with trends since 1990: a systematic analysis of progress, projections, and priorities*. PLoS Med 2011; 8: e1001080.
3. United Nations. *The Millennium Development Goals Report 2015*. New York, 2015.
4. Lawn J, Shibuya K, Stein C. *No cry at birth: global estimates of intrapartum stillbirths and intrapartum- related neonatal deaths*. Bull World Health Organ 2005; 83: 409–17.
5. Martines J, Paul VK, Bhutta ZA, et al. *Neonatal survival: a call for action*. Lancet; 365: 1189–97.
6. Lawn JE, Cousens S, Zupan J. *4 million neonatal deaths: when? Where? Why?* Lancet; 365: 891–900.
7. Lawn JE, Blencowe H, Pattinson R, et al. *Stillbirths: Where? When? Why? How to make the data count?* Lancet (London, England) 2011; 377: 1448–63.

**Plan of Investigation: Clinical observations and record keeping**

**Objectives**

1. To conduct and observational study to assess the current methods used for foetal and contraction monitoring (FCM) during labour.
2. To perform a criteria based audit to determine the adherence to current established MMH guidelines, and assess factors that influence adherence.
3. To determine challenges faced at MMH in FCM
4. To prepare for a validation study between observations and record books. (In addition to later compare this to previously analysed record keeping at the MMH in 2015.)

**Selection of labours**

Observations will be held at the maternity ward of MMH. At least 150 labours will be observed, maximum 8 labours per one shift. This corresponds to the shifts of the midwifes: morning shift: 7.30 am till 2.30pm, afternoon shift: from 2.30 pm till 8.30 pm and night shift: 8.30 pm till 7.30 am. Even though a labour exceeds the shift, observations will be continued until delivery. A schedule of which shift to incorporate will be created in advance in order to shadow as many midwives as possible, with different levels of experience. Labours will be selected based on the following criteria: the first women to reach active phase who meets the inclusion criteria and is either being admitted or already at the ward. The inclusion of labours will be both high- and low-risk (as determined antenatally or on admission), both nulli- and multipara. High-risk pregnancies are defined as women with a previous caesarean section, induced labour by oxytocin or having pregnancy, medical complications (preterm and postterm labours, PROM, meconium, maternal illness, severe bleeding, foetal distress (Intrauterine foetal death, IUFD), poor progress (crossed alert line of the partogram), severe hypertensive disorder, fever or other infections). Key events are defined as: maternal bleeding, temperature measurement (fever), fits, bleeding, caesarean section (CS), intra-uterine resuscitation, fluids etc. A labour will be observed once the woman reaches active phase of labour (4 cm cervical dilatation) until complete delivery of the baby (independent on whether the cervical dilatation is reached at admission or during intra-facility care). In case of an IUFD detected during labour, observations will be stopped, but data will be included in the results. Exclusion criteria for labour**:** woman for elective CS, diagnosed with IUD by staff before admission by ultrasound (US) or women who are send for immediate CS upon arrival. An information sheet in Kiswahili and a verbal explanation by a midwife in Kiswahili will be provided to the labouring women on the aim of this study: observing staff taking care of her. Informed consent will be asked from the labouring women (on arrival) and from the staff as a group (once study commences one time). Staff can tell upfront in case they do not want to participate. As an observer, my job is to observe the intrapartum care and I am not expected to intervene.

**Data collection tool**

A criteria based audit will allow us to compare current practice to the local standards. This involves structurally observing intrapartum care and determining the adherence to the local PartoMa guidelines. Therefore, a data collection is created, based upon the PartoMa guidelines and additional information on the labouring women to categorize the labours and to systematically note the observations and key events during each shift and labour is included.

**Data collection:** The gestational age (GA) will be calculated based on the available information: using the last menstrual period (LMP), ultrasound (US) findings from first trimester, fundal height measured in the first trimester or fundal height recorded on admission. This information will be collected after delivery. Please refer to supplement 1 for further details on data collected during clinical observation.

**Recordkeeping:** For now, I will solely prepare for the validation study, but photocopying the file and systematically saving it**.** Each file will have a number assigned to it and names will be covered. The timing of the recordkeeping will depend on the mode of delivery due to the loss of records at the MMH. A vaginal delivery can be recorded immediately after delivery and a CS after the morning meeting the next day latest. Method to collect data: KoBoToolbox.

**Data analysis**

PartoMa guidelines will be used to assess whether FCM was done according to the standards set of criteria we have assessed. The relevant criteria are described in the PartoMa guidelines. The influence of the following factors on adherence will be assessed: low-vs high-risk pregnancies, experienced vs non-experienced (self-identified), midwives or doctors, nulli- vs multipara, morning vs afternoon vs night shifts, occurrence of key vs non key events will be calculated:

1. Participant characteristics will be descriptively presented using means and standard deviations for continuous variables and frequencies and percentages for categorical variables.
2. FHM adherence according to standard will be calculated:
   - Percentage of number of labours that completely adhered to guidelines (dichotomized outcome, yes/no) for the following 3 groups: heart rate monitoring, contraction monitoring and both.
   - Score of FHM adherence, defined as: (number of observations) / (number of expected observations given the duration of labour).
   - Score of contraction monitoring adherence, defined as: (number of observations) / (number of expected observations given the duration of labour).
   - Score of maternal pulse adherence, defined as: (number of observations) / (number of expected observations given the duration of labour
   - Average interval between observations: average in minutes and standard deviation
3. Influence of low-vs high-risk pregnancies, experienced vs non-experienced midwives or doctors, nulli- vs multipara, morning vs afternoon vs night shifts, occurrence of key vs non key events on the FHM score will be assessed, using a Poisson distribution analysis.
4. Descriptive analysis of structural challenges and woman-to-staff contact.
