# Supplementary material for: Quality of intrapartum care: direct observations in a low-resource tertiary hospital
Source: Reprod Health. 2020 Mar 14;17:36. doi: 10.1186/s12978-020-0849-8 (PMC7071714; doi:10.1186/s12978-020-0849-8)
Supplement: Supplementary file 2 — Additional file 2. Definition of various terms used in the study [file 12978_2020_849_MOESM2_ESM.docx]

| **Definitions of specific terms in the study** | |
| --- | --- |
| **Term** | **Definition** |
| Skilled birth attendant | |
| Nurse-midwives | The term is used to describe both general nurses and midwives who provided skilled birth attendance in this setting. |
| Resident doctors | Medical officers (non-specialist) who worked exclusively in the department of obstetrics and gynaecology |
| Intern doctors | Newly graduated medical doctors undergoing their 12-week clinical rotation in the department of obstetrics and gynaecology |
| Senior doctors | Obstetrician/gynaecologist or medical doctor who have many years of experience in obstetrics. They act as second on duty/oncall in case residents need them for consultations and emergencies |
| WHO Partograph | A graphic record of labour progress (i.e. cervical dilatation) and parameters of maternal and foetal wellbeing. It includes parallel lines four hours apart (alert and action lines) to assist detection of abnormal labour progress. |
| Phases of labour | |
| First stage active phase | 4-10cm cervical dilation |
| Second stage | Full cervical dilatation (10cm) to delivery of the baby |
| Shift of inclusion | |
| Morning | 7:30-14:29 |
| Evening | 14:30-20:29 |
| Night | 20:30-07:29 |
| Gestational age | Length of pregnancy in weeks as determined by either last menstrual period or ultrasound during prenatal period. |
| Foetal heart rates (FHR) as defined by PartoMa guidelines version 1.2 | |
| Normal | 120-160bpm |
| Non-reassuring | 161-180 or 100-119bpm |
| Abnormal | >180 or <100bpm |
| Examination intervals | |
| FHR intervals | Minutes between two consecutive FHR examinations |
| VE intervals | Minutes between two consecutive vaginal examinations |
| Last FHR to delivery interval | Minutes between last FHR measured and delivery of the baby |
