# Supplementary material for: Quality of intrapartum care: direct observations in a low-resource tertiary hospital
Source: Reprod Health. 2020 Mar 14;17:36. doi: 10.1186/s12978-020-0849-8 (PMC7071714; doi:10.1186/s12978-020-0849-8)
Supplement: Supplementary file 3 — Additional file 3. Comparison of foetal and contraction monitoring recommendations in international, national and local (PartoMa) guidelines [file 12978_2020_849_MOESM3_ESM.docx]

| **Comparison of foetal and contraction monitoring recommendations in international, national and local (PartoMa) guidelines** | | | | |
| --- | --- | --- | --- | --- |
|  | | **Intermittent Auscultation** | | **Contractions** |
| **Guideline** | **Risk status** | **Frequency** | **Timing and duration** | **Timing and duration** |
|  |  |  |  |  |
| FIGO, 2015^1^ | Low | *1st Stage (active phase):*  15 minutes  *2nd Stage:*  Every 5 minutes | During and at least 30 seconds after contraction  At least 60 seconds | Before and during FHR auscultation, in order to detect at least two contractions  For 10 minutes |
|  | High | Continuous EFM | | |
| WHO IMPAC, 2000^2^ |  | *1st Stage (active phase):*  Every 30 minutes  2nd Stage:  Every 5 minutes | After contraction  For 60 seconds minute | Every half hour minutes  For 10 minutes |
| NICE, 2014, UK^3^ | Low | *1st Stage (active phase):*  At least every 15 minutes  *2nd Stage:*  At least every 5 minutes | After contraction At least 1 minute | Half hourly |
|  | High | Continuous EFM | | |
| PartoMa guidelines, 2015, Mnazi Mmoja Hospital^4^ | Normal FHR(120-160bpm) | *1st Stage (active phase):*  Every 30 minutes or every one hour as minimum  *2nd Stage:*  Every 15 minutes before pushing and after every contraction when pushing | After contraction for one minute  Assure not maternal pulse | 1st Stage (active phase):  Every 2 hours for 10 minutes  2nd Stage:  Every 30 minutes |
|  | Non-reassuring FHR (161-180 or 100-119bpm) | Every 15 minutes |  |  |
|  | Abnormal FHR (>180 or <100bpm) | If no improvement to >100bpm after 5 minutes, expediate delivery |  |  |
| References:  ^1^Lewis D, Downe S. FIGO consensus guidelines on intrapartum fetal monitoring: Intermittent auscultation. Int J Gynecol Obstet 2015;131:9–12  ^2^WHO, Managing Complications in Pregnancy and Childbirth: A guide for midwives and doctors, 2000  ^3^National Institute for Health and Clinical Excellence. Intrapartum care for health healthy women and babies. NICE 2014:1–58.  ^4^Maaløe N, Housseine N, Roosmalen J Van, Bygbjerg IC, Tersbøl BP, Khamis RS, et al. Labour management guidelines for a Tanzanian referral hospital : The participatory development process and birth attendants ’ perceptions. BMC Pregnancy Childbirth 2017;17:1–11. CE 2014:1–58. | | | | |
