# Supplementary material for: Quality of intrapartum care: direct observations in a low-resource tertiary hospital
Source: Reprod Health. 2020 Mar 14;17:36. doi: 10.1186/s12978-020-0849-8 (PMC7071714; doi:10.1186/s12978-020-0849-8)
Supplement: Supplementary file 4 — Additional file 4. Data collection sheets [file 12978_2020_849_MOESM4_ESM.docx]

Data Collection Tool – Per Shift

Date: …………

**Study title:** Foetal Heart Monitoring In LMIC: Novel Strategies for Better Perinatal Outcomes.

**Name of Organisation**: Mnazi Mmoja Hospital, Zanzibar; University Medical Centre Utrecht, Netherland

Shift: Morning/Evening/Night

| **Instrument** | | | Total No | | | | | | | | No currently functioning | | | | | | | | | Comments | | | | | | | | |
| --- | --- | --- | --- | --- | --- | --- | --- | --- | --- | --- | --- | --- | --- | --- | --- | --- | --- | --- | --- | --- | --- | --- | --- | --- | --- | --- | --- | --- |
| Pinard | | |  | | | | | | | |  | | | | | | | | |  | | | | | | | | |
| Foetoscope | | |  | | | | | | | |  | | | | | | | | |  | | | | | | | | |
| Doptone / Doppler | | |  | | | | | | | |  | | | | | | | | |  | | | | | | | | |
| Ultrasound | | | Mobile: yes / no Static: yes / no | | | | | | | | | | | | | | | | |  | | | | | | | | |
|  | | | | | | | | | | | | | | | | | | | | | | | | | | | | |
| **Staff** | Labour and delivery room (hourly) | | | | | | | | | | | | | | | | | | | | | | | | Comment (where if outside + doing what) | | | |
| Nurse |  | | |  | |  |  | |  | | |  | |  |  | |  | |  | | |  |  | |  | | | |
| Intern |  | | |  | |  |  | |  | | |  | |  |  | |  | |  | | |  |  | |  | | | |
| Doctor |  | | |  | |  |  | |  | | |  | |  |  | |  | |  | | |  |  | |  | | | |
| Student |  | | |  | |  |  | |  | | |  | |  |  | |  | |  | | |  |  | |  | | | |
| Senior /  Outsiders |  | | |  | |  |  | |  | | |  | |  |  | |  | |  | | |  |  | |  | | | |
|  | | | | | | | | | | | | | | | | | | | | | | | | | | | | |
| **Women** | | **Morning** | | | | | | | | | | | | | | | | **Other** | | | | | | | | | | |
|  |  | Labour room | | | | | | | | Caesar | | | | | | | | Postnatal 1 | | | | | | | | Postnatal 2 | | |
| Labour | |  | | |  | | |  | |  | | |  | | |  | |  | | |  | | |  | |  |  |  |
|  |  |  | | |  | | |  | |  | | |  | | |  | |  | | |  | | |  | |  |  |  |
| Postnatal | |  | | |  | | |  | |  | | |  | | |  | |  | | |  | | |  | |  |  |  |
|  |  |  | | |  | | |  | |  | | |  | | |  | |  | | |  | | |  | |  |  |  |
| Non-labour | |  | | |  | | |  | |  | | |  | | |  | |  | | |  | | |  | |  |  |  |
|  |  |  | | |  | | |  | |  | | |  | | |  | |  | | |  | | |  | |  |  |  |
| **Legend:** Morning: 8, 10, 12, 14; Evening: 15, 17, 19, 21; Night: 20, 22, 0, 2, 4, 6 o’clock | | | | | | | | | | | | | | | | | | | | | | | | | | | | |
| **Other factors of influence:** (e.g. emergency: note time what time occurred) | | | | | | | | | | | | | | | | | | | | | | | | | | | | |

Data Collection Tool – Per Patient

Age:......... G: …….. Parity:........ Living:...... Stillbirths:........ Neonatal deaths:.......... GA:............ EFW: ......... No. of antenatal visits: …

**Known medical and surgical illnesses:**

**Complications during previous pregnancies and after delivery:**

**Complications this pregnancy:** Anaemia, Hypertensive disorder, Diabetes, Previous CS, Premature labour, Placenta praevia, PV bleed, Fever, Prolonged ROM

| **Nr.** | **Time** | **Staff** (nurse /int/ doc/ stud) | **VE** (+/-) | **FHR** (+/-) | **Method** (DL/ US/ Dop/P) | **Timed** (+/-) | **Mat. pulse** (+/-) | **Screen used?** (+/-) | **Action taken/ Events** (e.g. fluids, injections, CAD, decision for CS, BP, going to delivery room, calling for help, oxytocin, epi, bleeding) | **Commun after?** (+/-) | **Rec. on graph** (+/-) | **Who is around during exam?** | **Contrac.**  **Mon (+/-)** |
| --- | --- | --- | --- | --- | --- | --- | --- | --- | --- | --- | --- | --- | --- |
| 0 | Date / Time/ Dilatation at admission: | | | | | | | | | | | | |
| 1 | : |  |  |  |  |  |  |  |  |  |  |  |  |
| 2 | : |  |  |  |  |  |  |  |  |  |  |  |  |
| 3 | : |  |  |  |  |  |  |  |  |  |  |  |  |
| 4 | : |  |  |  |  |  |  |  |  |  |  |  |  |
| 5 | : |  |  |  |  |  |  |  |  |  |  |  |  |
| 6 | : |  |  |  |  |  |  |  |  |  |  |  |  |
| 7 | : |  |  |  |  |  |  |  |  |  |  |  |  |
| 8 | : |  |  |  |  |  |  |  |  |  |  |  |  |
| 9 | : |  |  |  |  |  |  |  |  |  |  |  |  |
| 10 | : |  |  |  |  |  |  |  |  |  |  |  |  |
| 11 | : |  |  |  |  |  |  |  |  |  |  |  |  |
| 12 | : |  |  |  |  |  |  |  |  |  |  |  |  |
| 13 | : |  |  |  |  |  |  |  |  |  |  |  |  |
| 14 | : |  |  |  |  |  |  |  |  |  |  |  |  |
| 15 | : |  |  |  |  |  |  |  |  |  |  |  |  |
| 16 | : |  |  |  |  |  |  |  |  |  |  |  |  |
| Outcome: SVD/ CS/ Vacuum Time start of second stage: / Unclear Time of delivery: Place of delivery: Labour or Delivery or Theatre Birth Weight: Apgar score: at 1/5 min: / Sex: Male / Female, NICU admission: Yes / No, Neonatal Death: Yes / No, Resuscitation: Yes / No Total people who provided her care: | | | | | | | | | | | | | |
| How long was midwife present during delivery? Meconium (when?):  Summary of delivery:  Other comments: | | | | | | | | | | | | | |
| **Legend**: + = done, - = not done DL = DeLee, Dop = Doppler, P = Pinard, US = ultrasound, CAD = catheter, BP = blood pressure, Epi = episiotomy | | | | | | | | | | | | | |
