# Supplementary material for: Quality of intrapartum care: direct observations in a low-resource tertiary hospital
Source: Reprod Health. 2020 Mar 14;17:36. doi: 10.1186/s12978-020-0849-8 (PMC7071714; doi:10.1186/s12978-020-0849-8)
Supplement: Supplementary file 5 — Additional file 5. Predictors of the frequency of FHR monitoring [file 12978_2020_849_MOESM5_ESM.docx]

| **Predictors of the frequency of FHR** monitoring** | | | | |
| --- | --- | --- | --- | --- |
|  | **Univariate regression** | | **Multivariate regression** | |
|  | **Rate Ratio**  **(Confidence Interval)** | **P-value** | **Rate Ratio (Confidence Interval)** | **P-value** |
| **Expected number of FHR monitoring** | 1.85 (1.64-2.09) | <0.0001* | 1.76 (1.54-2.01) | <0.0001* |
| **Risk category (low/high)** | 1.15 (0.96-1.38) | 0.119 | 1.11(0.93-1.33) | 0.249 |
| **Shift of admission** |  |  |  |  |
| **Morning** | Ref | Ref | Ref | Ref |
| **Evening** | 1.11 (0.89-1/39) | 0.342 | 1.10 (0.88-1.35) | 0.438 |
| **Night** | 0.98 (0.79-1.21) | 0.860 | 0.95 (0.77-1.15) | 0.560 |
| **Parity (Nulli-/multi-parity)** | 1.08 (0.90-1.29) | 0.424 | 1.00 (0.83-1.21) | 0.77 |
| **Occurrence intrapartum risk events (yes/no)***** | 1.37 (1.14-1.65) | 0.001* | 1.32 (1.09-1.58) | 0.004* |
| **Meconium** | 1.28 (1.02-1.60) | 0.032* |  |  |
| **Oxytocin** | 1.30 (1.07-1.58) | 0.008* |  |  |
| **Non-reassuring/ abnormal FHR** | 1.68 (1.26-2.24) | 0.0004* |  |  |
| **Fever** | 1.45 (0.83-2.52) | 0.189 |  |  |
| **Action line Crossed** | 1.21 (0.97-1.52) | 0.0913 |  |  |
| * p< 0.05 = statistically significant.  ** FHR = foetal heart rate  *** Occurrence of either Meconium, Oxytocin, Non-reassuring/abnormal FHR, Fever, Action line Crossed. Rate ratio for these individual intrapartum risk events (empty fields) are given in the text because they were derived from a separate multivariate analysis. | | | | |

**Additional file 5**
